# The Place of Surgery in Hyperthyroidism
*Abridged from an address delivered to the Bristol Medico-Chirurgical Society on Wednesday, February 14th, 1934.


**Published:** 1934

**Authors:** Thomas P. Dunhill

**Affiliations:** Honorary Surgeon to H.M. the King; Associate Surgeon, Professorial Unit, St. Bartholomew's Hospital


### The Bristol Medico-Chirurgical Journal

" *Scire est nescire, nisi id me*
*Scire alius sciret.*"

AUTUMN, 1934.

### THE PLACE OF SURGERY IN HYPERTHYROIDISM.*

BY

Sir Thomas P. Dunhill, K.C.V.O., F.R.A.C.S.,

*Honorary Surgeon to H.M. the King;*
*Associate Surgeon, Professorial Unit, St. Bartholomew's Hospital.*

During the last twenty years a great change has come over our views on this subject. In a collection of all the cases of Graves's disease treated medically in Guy's Hospital between 1890 and 1910 there were 169 patients, of whom 18 died in hospital; and of 49 patients traced after discharge a further 8 had died within three years. In St. Thomas's Hospital 34 cases were treated surgically between 1911 and 1914[1]; of 15 cases treated by arterial ligation 3 died

* Abridged from an address delivered to the Bristol Medico-Chirurgical Society on Wednesday, February 14th, 1934.

(*i.e.* 20 per cent.), while of 19 hemithyroidectomies 8 died (42 per cent.). These two publications give us a reasonable picture of the position at that time.

At the present day medical treatment is on a surer foundation : X-ray treatment is more ably controlled ; and for those who fail to respond sufficiently to these measures operation may be contemplated without undue fear, and a level of health regained which previously could scarcely have seemed possible. This has been brought about by gradual stages, which it is scarcely necessary now to traverse. It was felt that whatever was the ultimate nature of the condition the thyroid was responsible for its manifestations. Selection of cases for operation began to lessen the operative risk, as also the realization that this cannot be treated as an emergency operation. And experience with various anæsthetics soon led to chloroform being abandoned. Local anæsthesia helped greatly, and then nitrous oxide quickly displaced ether. The death-rate had already dropped considerably before Plummer popularized and standardized treatment by iodine, and patients were being operated on with reasonable safety who a few years earlier would have been excluded as unsafe risks.

In our profession there will always be individuality, and it is right that there should be. Progress comes by individuals with vision working, thinking, testing their theories, accepting what proves to be good and building it into the structure of our knowledge. In one period of time there will be progress along medical lines—iodine, elimination of sepsis and improvement in environment, using that term in its widest sense. In another period of time improvement in technique will have become apparent, or in pre-operative care, in grading the operation to the patient's strength, or in saving multiple operations by achieving in one stage the maximum compatible with safety. In this way, here a little, there a little, sometimes working individually, but always really in co-operation and with knowledge of what our co-workers are doing, we serve the interests of our patients and give our contributions to the sum of medical knowledge.

There is no hard-and-fast line between medical and surgical treatment of Graves's disease. It will be accepted, I think, by all physicians that surgery has some place, and by all surgeons that medical management is of paramount importance. The young patient or any patient seen early belongs to the family practitioner and the physician, whose function it is to bring about a restoration of equilibrium. For how many persons nearly get this disease, but just don't, and how many, if they are seen just after they have become definitely afflicted, could be saved by some alteration of environment ?

I propose for the moment to consider some of the less usual aspects of this disease, aspects nevertheless which any of us from time to time may have to meet and which some of us are certainly meeting all the time.

CHILDREN.—In the years 1911–1914 6 patients died in England and Wales from the disease under the age of 5 years, and 47 under the age of 15.[1] Of my own cases under the age of 15, 8 in number, 2 died, 2 were successfully treated by X-rays, 2 by arterial ligation, and 2 required operative resection of both lobes. I have tried to conserve the gland and to operate only if it is imperilling the life of the child. If that stage is reached the child must be treated on the same lines as the adult, but ever inclining to leave more gland than in the adult cases—the operation must be what is just adequate to save life, but if any degree of hypothyroidism were induced it is possible that physical or mental development might be impared. Those who are a little older may not feel ill and may decline to acknowledge that they are ill; they resent treatment, and even when actually feeling ill refuse to give the time necessary for medical treatment, but demand operation unnecessarily.

CARDIAC COMPLICATIONS.—The heart feels the impact of the disease on its neuromuscular mechanism, and the response differs according to whether it is a young heart or an elderly heart. Among my cases auricular fibrillation occurred in 231 cases. Of these surgical treatment restored a regular rhythm in 102 cases without other measures, but in a further 81 cases quinidine was needed in addition before a regular rhythm was achieved. The death-rate among these patients has proved to be 7 per cent. in contrast to the 2·7 per cent. for the whole series.

GLYCOSURIA.—One is disposed to speculate when this complication occurs whether it is due to the pancreas being specifically affected as part of the disease, whether there is a general disturbance of the whole endocrine system, or whether the glycosuria is due to the pituitary being affected. In these cases there is no doubt that medical treatment should be tried first. It may be possible to control the glycosuria although the sugar threshold remains low. But if the condition progresses so that the glycosuria is no longer controlled by insulin operative treatment becomes imperative. Of 49 patients in my series with this complication 2 had severe auricular fibrillation as well; one of these is well after operation, but still requires insulin; another without fibrillation remains well eight years after his operation.

EMACIATION.—This very troublesome complication is due, obviously, to the high basal metabolism associated with the disease. One of my patients weighed 4 st. 1 lb. at the time when we were compelled to operate (normal 8 st. 7 lb.). Another was normally 9 st., on admission 5 st. 1 lb.: when he had wasted to 4 st. 7 lb. we had to operate. Three others had lost five stone and two four stone in weight. These are all patients who could not be made to regain weight in hospital or nursing home under able advice and for lengthened periods. In operating on these cases it is my custom to ligate the superior arteries first; this may render it safe to excise the lobes singly or together, but often it is necessary to ligate the inferior arteries also before this can be undertaken.

MENTAL CHANGES.—Quite early there may be a change in temperament with a loss of tranquillity leading to loss of mental balance. These patients may get steadily worse until actual mental derangement occurs. In these cases operative treatment is urgently necessary. 12 of my patients showed severe mental derangement, 10 being in asylum or mental home. Of these, following operation, 7 recovered completely, 3 did not, 1 died after the operation. In the twelfth case all thyroid symptoms disappeared, but the mental condition took the form of fixed suicidal ideas, and she subsequently actually committed suicide. In spite of the two cases last mentioned, the balance is overwhelmingly in favour of operation to lessen the toxicity of the disease and thus restore tranquillity, but be absolutely sure that the mental condition is *due* to the hyperthyroidism.

EXCESSIVE RESTLESSNESS.—This I regard as a most dangerous sign. The patient is really in a crisis—medical, not surgical. Although frequently the surgeon is called on at this stage and pleaded with to operate, such a course is invariably fatal.

RESULTS OF TREATMENT.—As occurs with surgery in some other parts of the body, the claims made for operative treatment are sometimes unduly optimistic. The slogan in some quarters is: "Give iodine—and all are safe and all are cured." But all are *not* safe, and all are *not cured.* It would be idle as well as unjust to let it be assumed that this operation is carried through without risk, in spite of the great improvements in preparation of the patient and in technique of the operation. We see patients in every grade of illness, and still some are brought to hospital practically moribund. Others have suffered damage from the complications of which we have just been speaking, and which, as we have seen, may be very severe. But we are also justified in asking: "*Why* have they reached that condition without efficient help being given them previously?" One of the most pressing needs is to detect quickly when a patient just ceases to hold and first begins to slip.

But to come back to the desperately ill patient. For these patients with their reserves exhausted all our judgement is required in grading the amount we do to the patient's strength. A little too much, and we lose the patient; yet enough must ultimately be done to effect a cure. The patient may be greatly disappointed at multiple operations, but we cannot help that. We are not responsible for the disease, but it is our responsibility to save life and to restore the maximum degree of health possible.

Nor must patient and practitioner be allowed to assume that recovery is complete, and the patient placed on the level of health he might have enjoyed had he not suffered from this disease. Life may be his, but with limitations; the eyes may remain proptosed, capacity for work may be diminished, blood-pressure may rise, a few may not regain a normal cardiac rhythm.

Notwithstanding this, the results of operation are in a high percentage of cases extremely good, and in many are little short of miraculous. But there remain some cases where the capacity of the patient to respond has been lost. Some of these may never have been robust, apart from the disease, and some through years of illness have lost capacity to regain physical reserves. In a few again in whom we think we have a right to expect a high degree of recovery we are disappointed. This is not to disparage the extremely good results of operative treatment. During the eleven years 1921–1931 I have followed up 428 of my patients. 360 are able to lead approximately normal lives (84 per cent.), 37 still require medical guidance (8 per cent.), 18 are untraced, and 13 have died (3 per cent.). Because

I have insisted on the limitations we must not lose a proper sense of proportion, but must realize that in very many cases surgery can offer to the patient physical and mental tranquillity, a higher level of health, a higher plane of safety.

### REFERENCE.

[1] Sir Hector Mackenzie, Bradshaw Lecture, *Lancet*, 1916, vol. ii., pp. 815–821.

